# The Advances, Challenges, and Perspectives on Electrocatalytic Reduction of Nitrogenous Substances to Ammonia: A Review

**DOI:** 10.3390/ma16247647

**Published:** 2023-12-14

**Authors:** Liu Yang, Huichun Han, Lan Sun, Jinxiong Wu, Meng Wang

**Affiliations:** 1 Queen Mary University of London Engineering School, Northwestern Polytechnical University, Xi’an 710129, China; yangliu2022@mail.nwpu.edu.cn (L.Y.); hanhuichun@mail.nwpu.edu.cn (H.H.); lansun@mail.nwpu.edu.cn (L.S.); 2 University and College Key Lab of Natural Product Chemistry and Application in Xinjiang, School of Chemistry and Chemical Engineering, Yili Normal University, Yining 835000, China; 3 School of Materials Engineering, Xi’an Aeronautical University, 259 West Second Ring, Xi’an 710077, China

**Keywords:** electrocatalysis, ammonia, nitrogen, nitrate, two mechanisms

## Abstract

Ammonia (NH_3_) is considered to be a critical chemical feedstock in agriculture, industry, and other fields. However, conventional Haber–Bosch (HB) ammonia (NH_3_) production suffers from high energy consumption, harsh reaction conditions, and large carbon dioxide emissions. Despite the emergence of electrocatalytic reduction of nitrogenous substances to NH_3_ under ambient conditions as a new frontier, there are several bottleneck problems that impede the commercialization process. These include low catalytic efficiency, competition with the hydrogen evolution reaction, and difficulties in breaking the N≡N triple bond. In this review, we explore the recent advances in electrocatalytic NH_3_ synthesis, using nitrogen and nitrate as reactants. We focus on the contribution of the catalyst design, specifically based on molecular–catalyst interaction mechanisms, as well as chemical bond breaking and directional coupling mechanisms, to address the aforementioned problems during electrocatalytic NH_3_ synthesis. Finally, we discuss the relevant opportunities and challenges in this field.

## 1. Introduction

With the continuous development of industry and agriculture, the yearly increase in carbon dioxide (CO_2_) emissions has resulted in numerous environmental problems and has drawn extensive attention from the international community. Therefore, reducing the carbon footprint by optimizing chemical processes is of paramount importance [1]. Ammonia (NH_3_) is not only one of the most productive industrial chemicals worldwide, but also serves as the cornerstone of modern industrial and agricultural development [2,3]. For instance, NH_3_ can be used to manufacture various products, including fertilizers, explosives, plastics, synthetic fibers, and dietary proteins, among others, which dominate industrial applications [2,4]. However, conventional Haber–Bosch (HB) ammonia (NH_3_) production still faces significant challenges. The Haber–Bosch process involves reacting hydrogen and nitrogen over an iron-based catalyst at temperatures approaching 500 °C and pressures up to 300 bar to form ammonia [2]. On one hand, this process leads to the release of a large amount of carbon compounds, such as CO and CO_2_, during the production process, contributing to the severe climate crisis and extreme weather conditions (e.g., melting Arctic permafrost, large-scale coral bleaching, etc.) [5,6,7,8,9]. On the other hand, the smooth operation of this process heavily depends on massive fossil fuel combustion and substantial energy consumption. In reality, thermal–catalytic NH_3_ synthesis technology requires harsh reaction conditions (Figure 2). To overcome this dilemma, innovative strategies such as photocatalytic, electrocatalytic, and chemical looping approaches have been proposed for effective NH_3_ synthesis [10,11,12,13,14,15].

**Figure 1:**
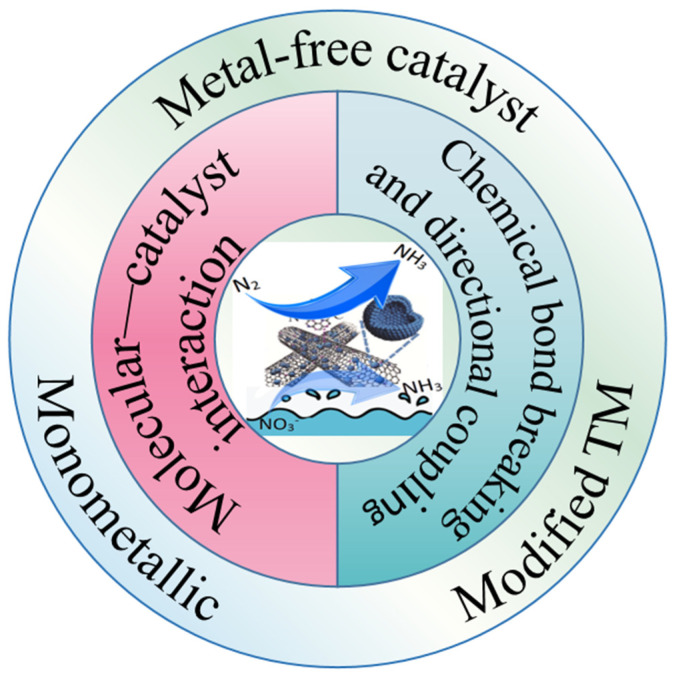
The main elements of electrocatalytic ammonia synthesis discussed in this review.

N_2_ and NO_3_^−^ are currently regarded as the most extensively used feedstocks for electrocatalytic NH_3_ synthesis. Although this reaction possesses excellent characteristics, there are several challenges in actual experiments, including the difficulty of N_2_ adsorption and activation, as well as the difficulty of directional coupling of products. However, through relentless efforts, scientists have identified solutions to these problems based on two mechanisms: the molecular–catalyst interaction, and chemical bond breaking and directional coupling. The molecular–catalyst interaction mechanism refers to the process in which a catalyst is added to the reaction system and interacts with the reactants to activate them or modify the reaction rate. The mechanism of chemical bond breaking and directional coupling, on the other hand, involves breaking the chemical bond of a reactant under certain conditions and forming a new chemical bond after a specific treatment to synthesize a desired substance. This review summarizes the current research status of electrocatalytic ammonia synthesis using nitrogen and nitrate, focusing on the two mechanisms of molecular–catalyst interaction and chemical bond breaking and directional coupling. Additionally, it presents new challenges in the electrocatalytic ammonia synthesis process.

## 2. Electrocatalyzed N_2_ Synthesis of Ammonia

### 2.1. Mechanism of Molecular–Catalyst Interaction for Electrocatalytic N_2_ Reduction Reaction

During electrocatalytic ammonia synthesis using nitrogen as the raw material, there is competition between the N_2_ reduction reaction (NRR) and the hydrogen evolution reaction (HER), as they have similar overpotentials. This competition results in a low Faraday efficiency (FE) of the reaction, leading to significant wastage of the raw materials and increased energy consumption [16,17,18]. To address this issue, researchers have made continuous efforts to find new catalysts that improve the selectivity of the main reaction through the mechanism of the molecular–catalyst interaction. However, experiments have shown that the use of classical iron catalysts leads to significant energy loss, necessitating modifications to the existing catalysts.

#### 2.1.1. Inhibiting the Competition from the Hydrogen Evolution Reaction

**Figure 3:**
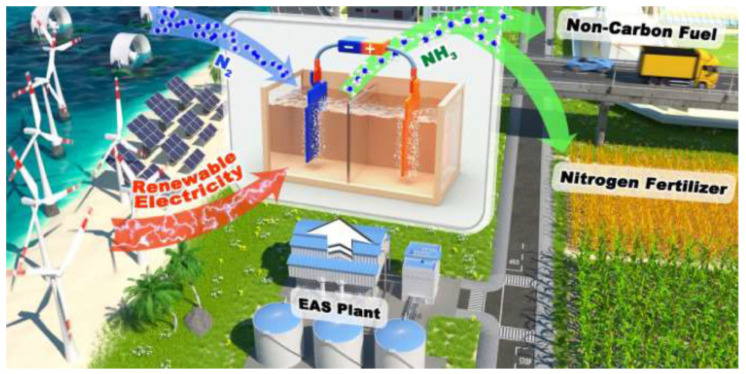
Illustration of the use of renewable electricity to drive electrocatalytic nitrogen synthesis of ammonia, and important applications of ammonia: carbon-free fuels (future) and nitrogen fertilizers. Copyright 2020, Elsevier.

Electrocatalytic systems, which utilize water as the hydrogen source instead of fossil fuels, often rely on renewable energy sources, such as solar, wind, and hydro power, to provide the necessary energy for the NRR (Figure 3) [19]. However, one of the main challenges in the NRR is that the HER is kinetically more favorable than the NRR, which negatively impacts the efficiency of the NRR and reduces ammonia production. Therefore, it is crucial to screen and design electrocatalysts with high catalytic activity and selectivity. Significant progress has already been achieved in this field [20].

**Scheme 1:**
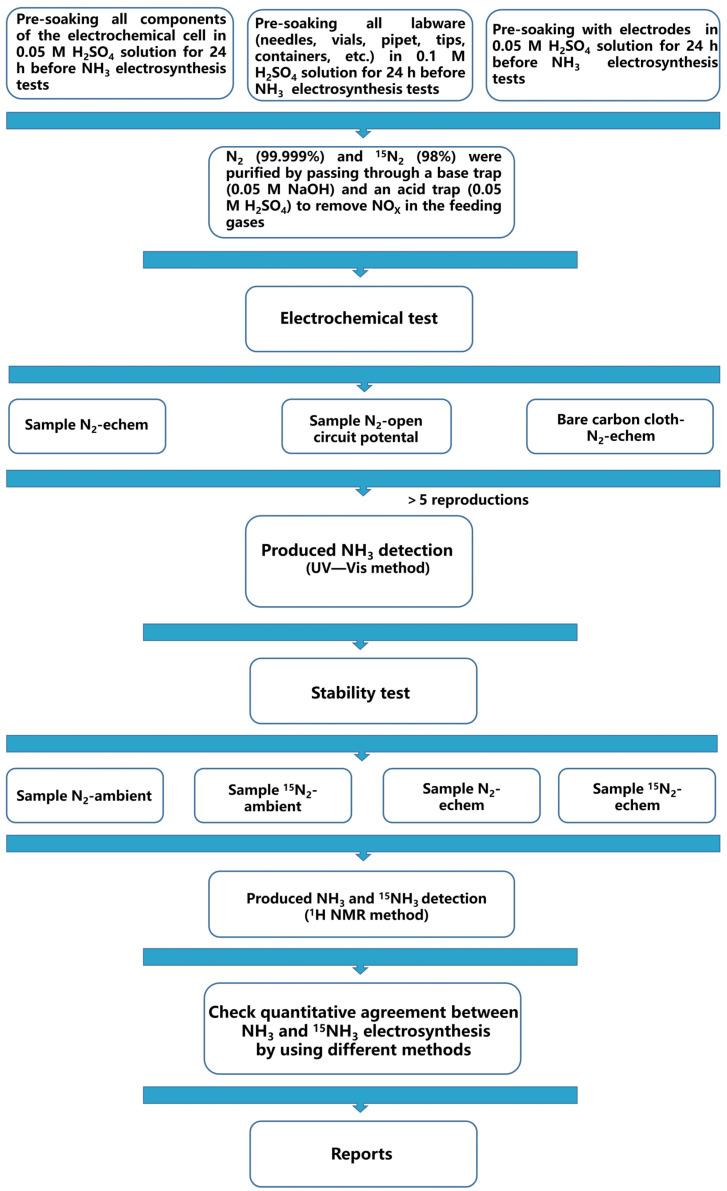
The NH_3_ electrosynthesis experimental procedures utilized for excluding potential contaminants.

Two types of catalysts, metallic and non-metallic, are currently the most extensively studied [16,21]. Metallic catalysts can be categorized as precious metal and non-precious metal catalysts. Electrochemical ammonia synthesis using precious metal catalysts is highly sensitive to the catalytic structure. The energy barrier for nitrogen dissociation depends largely on the particle size and crystal structure of the metal active center. For the NRR, a simple and effective method of electroplating rhodium and ruthenium on titanium felt using an electrochemical membrane reactor has been proposed. Linear sweep voltammetry confirms that Ru and Rh coatings can promote the electrochemical synthesis of NH_3_. Among the metal catalysts, Ru is particularly suitable for electrochemical NH_3_ synthesis due to its higher activity [22,23]. Additionally, a model combining a porous graphite carbon nitride matrix (Ru SAs/g-C_3_N_4_) has been suggested [24], which allows control over the reaction selectivity by adding an applied potential, thereby adjusting the local chemical environment and size of the active site of Ru. The highly dispersed Ru sites on the g-C_3_N_4_ substrate significantly enhanced the exposure of the catalytic active site and the emerged concerted interplay between the single-atom sites and the substrate regulating the electron configuration of the metal sites, which prompted the adsorption of the key intermediates and, thus, achieved a superior NRR performance. Due to the extremely low catalytic activity (typically in the order of micrograms), traces of NO_x_ pollutants in the atmosphere may cause false-positive results in the above reactions. Therefore, for N-containing catalysts, it is critical to eliminate possible interference with the reaction results from airborne contaminants and the catalyst itself. The authors used ^15^N_2_ isotope labelling experiments to demonstrate that the N in NH_3_ indeed originates from N_2_ and not from other pollutants. As electrocatalytic NRR has received more attention from researchers, it is particularly important to establish a rigorous reaction process to exclude interference from airborne pollutants and the catalyst itself. As delineated in Scheme 1, each separate component of the electrolyte solution (e.g., electrolyte salt, solvent, and electrolyte solution) should be confirmed that it is not polluted by other forms of contamination. Besides, all the glassware, electrodes, and labware were kept inside a sealed electrochemical cell, with no potential applied for 24 h to exclude possible contamination processes. Rigorous gas purification processes and electrocatalytic tests (including the setting up of multiple comparative and repetitive experiments, as well as the cross-checking of product yields using different test methods) are indispensable to ensure the rigor of the electrocatalytic nitrogen reduction reaction. Monatomic catalysts, although limited in the size of the reduction, offer nearly 100% metal dispersion, and they are usually the active sites for the electrochemical reduction of nitrogen and are able to form stable chemical bonds with nitrogen atoms, which facilitates the breaking of the N≡N bond [25]. It enables them to exhibit good activity and selectivity. However, the scarcity and high cost of precious metal catalyst materials hinders their large-scale industrial application. To overcome this limitation, it is important to explore low cost and abundant alternative materials, such as non-precious metals, and conduct research to enhance their electrocatalytic performance as potential substitutes for precious metal catalysts. For example, Mo has been widely studied as a non-noble metal catalyst in the electrochemical synthesis of ammonia due to its excellent electrocatalytic performance [22,24]. Non-precious metal catalysts, while overcoming the high-cost challenge, face the problem of low FE [26]. Metal catalysts are d-orbital catalysts, whereas non-metal catalysts activate inert N_2_ molecules through sp hybrid orbitals, and this difference in the activation mechanism also renders the non-precious metal NRR catalysts more suitable for the N_2_ molecules. The different activation mechanisms also make the non-metallic catalysts valuable for research.

In recent decades, there has been a proliferation of metal-free catalysts [27]. Researchers quickly realized the potential benefits of these catalysts and have begun making various modifications to make them more widely used in place of traditional metal-based catalysts. Based on the characteristics of the NRR, it is crucial to find metal-free catalysts with abundant active sites to enhance the adsorption and activation of N_2_. Carbon-based materials doped with N and P have shown promise as highly active materials suitable for NRR due to their excellent electrical conductivity and durability [28]. The surface of defective carbon materials provides unsaturated coordination sites for the chemisorption of N_2_. For example, in 2018, scientists combined a metal-free polymer, carbon nitride, with abundant Nvs. (PCN-NVs) to act as a highly active nitrogen reduction reaction (NRR) catalyst in order to significantly enhance the ammonia (NH_3_) yield and FE. The PCN-Nvs. demonstrated excellent N_2_ adsorption and activation capabilities due to the interconnectedness of the materials, resulting in exceptional properties. The experimenters used isotopic labeling in this experiment to demonstrate that all the NH_3_ detected in the results came from the supplied N_2_, eliminating all possible contamination from the N-containing catalyst and ensuring the accuracy of the results [16,29].

**Figure 4:**
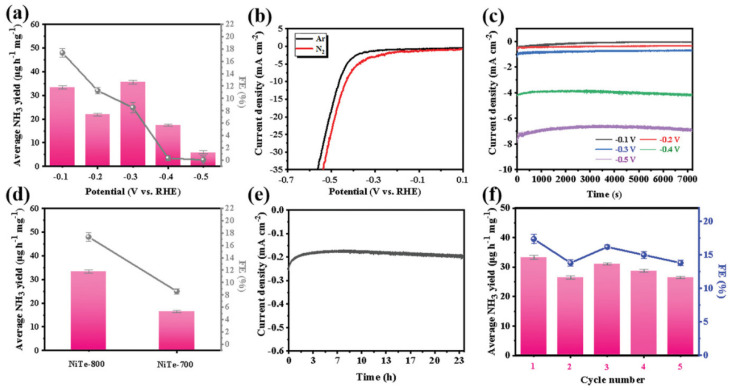
(**a**) FE and NH_3_ yield rate for NiTe-800 within the potential, ranging from −0.1 to 0.5 V. (**b**) LSV curves for NiTe-800. (**c**) The chronoamperometric curves for NiTe-800 at various potentials for 2 h in HCl solution. (**d**) FE and NH_3_ yield rate for NiTe-700 and NiTe-800. (**e**) The chronoamperometric curves for NiTe-800 for 24 h in HCl solution. (**f**) FE and NH_3_ yield rate for NiTe-800 at −0.1 V during recycling tests repeated five times. Copyright 2020, John Wiley and Sons.

Over time, considering the good performance of Ni-based catalysts for NRR, researchers began to explore the design and synthesis of Ni-based catalysts with certain exposed surfaces, such as nickel telluride (NiTe) nanocrystals [30,31]. By employing a straightforward synthesis process, scientists were able to selectively expose the {001} and {010} surfaces of NiTe nanocrystals. This precise atomic-level manipulation of the surface chemistry allowed for high selectivity in the NRR process. Remarkably, the scientists successfully designed the surface atomic structure of NiTe nanocrystals to achieve efficient NRR at 0.1 μm HCl. The results demonstrated that NiTe nanocrystals with {001} surface exposure exhibited superior NRR performance compared to those with {010} surface exposure (Figure 4). This enhanced activity can be primarily attributed to the optimized surface atomic arrangement of the exposed {001} surface, which allowed for efficient electrical reduction of N_2_ to NH_3_ through an alternating mechanism. On the contrary, {010} surfaces, consisting of a single Te bit, had a surface structure that obstructed the NRR process, resulting in poor performance. Engineering the surface atomic architecture of the catalyst to selectively expose the {001} facets guaranteed the adsorption and activation of N_2_ and weakened the binding for *H (* means catalytically active site) species. This work provides valuable guidance for the expansion and enhancement of catalyst properties [30,31].

However, when selecting catalysts to inhibit the hydrogen evolution reaction (HER), it is important to note that uncontrolled HER inhibition may not be a reasonable strategy for developing an effective NRR catalyst. From the point of view of the reaction mechanism, NRR is a process in which N_2_ reacts with protons and electrons to form NH_3_. This process can be briefly summarized in three stages: (1) the protons acquire electrons on the catalyst surface, thus H + e^−^ + * = H*; (2) activation of N_2_ occurs in which gaseous N_2_ reacts with surface H* species to form N_2_H_2_*, thus N_2_(g) + 2H* = N_2_H_2_*; (3) after the formation of N_2_H_2_* species, protons and electrons will keep attacking the N_2_H_2_* group to produce two NH_3_ molecules. The reactions in this process are all exothermic, so it is known that N_2_H_2_ will not appear as a final product, according to reaction kinetics and thermodynamics [32]. In this scenario, HER plays a dual role in the electrocatalytic NRR process. On one hand, the HER competes with the NRR, leading to decreased ammonia yield and FE. On the other hand, excessive HER inhibition can hinder the hydrogenation of nitrogen (N), since an appropriate amount of hydrogen (H) is required in the electrolyte for N_2_ protonation. In theory, if a catalyst cannot activate protons, it will struggle to hydrogenate N≡N. Molybdenum (Mo), cobalt (Co), and platinum (Pt)-based materials are known to exhibit HER activity. Recent studies have shown that utilizing these materials as catalysts for NRR has achieved relatively high NRR performance, surpassing the abovementioned catalysts that simultaneously inhibit the HER and promote the NRR [33,34].

#### 2.1.2. Retrofit Existing Catalysts to Reduce Energy Consumption

Most commercial plants currently employ multi-catalyzed iron-based catalysts; however, this process requires high temperatures and pressures, leading to significant energy consumption. In light of this, numerous researchers have reported novel active catalysts for ammonia synthesis under milder conditions. Following the groundbreaking report by Aika et al. on the highly active Ru catalyst for ammonia synthesis, the scientific community has directed its attention towards Ru catalysts. Nevertheless, Ru catalysts are associated with two key challenges: high cost and carbon support methanation in harsh industrial conditions. Consequently, scientists continue to search for improved catalysts. In the process, researchers have found that promising catalysts for ammonia synthesis can be obtained by modifying transition metal elements [35,36].

##### Transition Metal Catalysts Using Nanomaterials as Carriers

As an example, previous investigations have revealed that cobalt (Co) exhibits a certain degree of nitrogen adsorption ability, and Co/Ce_0.5_Zr_0.5_O_2_, a Co-containing catalyst effectively facilitate N_2_ dissolution, making them suitable for ammonia synthesis reactions [37]. The catalytic activities of the Co and Co/Ce_0.5_Zr_0.5_O_2_ were calculated using a linear combination of fits with the Athena software (1.28.4), with the former showing a catalytic activity of 174.6 µmol g^−1^ h^−1^ and the latter showing a catalytic activity of 293.5 µmol g^−1^ h^−1^. These results indicate that the modified Co-containing catalysts have good catalytic activities [38]. Although Co is not cheaper than Ru, ammonia can be synthesized at lower temperatures using Co catalysts in the presence of an electric field, thus reducing industrial production costs [37,38,39]. Moreover, transition metal-embedded carbon nanotubes (M@NCNTs) possessing the Mott–Schottky feature promoted spontaneous electron transfer between the different domains and elevated the Fermi energy levels of the outer carbon nanotubes and the C-N (π) orbitals formed with N_2_ molecules, accelerating the electron transfer from the catalyst to the N_2_ molecule and leading to inert N≡N triple bond breakage [30].

##### Transition Metal Complexes

As early as 1965, Allen and Senoff discovered the transition metal N_2_ complex and pioneered the preparation of the first transition metal N_2_ complex (Ru(NH_3_)_5_(N_2_)]^2+^) [40]. Since then, transition metal complexes have shown high value in the academic field. However, there have been few reports until 2003 on the reduction of nitrogen to ammonia catalyzed by transition metal complexes, when Yandulov and Schrock successfully synthesized the chemistry of Mo complexes that contain a triamidoamine (((ArNCH_2_CH_2_)_3_N)^3–^, which is (ArN_3_N)^3−^, where Ar is aryl) ligand. Experimental results demonstrated that the most successful efficiency of this class of chemicals in catalyzing the reduction of nitrogen to ammonia (63% to 66%) is second only to that of the highest known catalytic efficiency of Fe/Mo nitrogenase (75%) [41]. Until today, transition metal complexes catalyzing NRR have been the focus of research, such as Rb_2_(Mn(NH_2_)_4_), K_2_(Mn(NH_2_)_4_), and transition metal-LiH composite catalysts, which have been shown to effectively break the scaling relations and achieve ammonia synthesis under mild conditions. Because they exhibit two temperature-dependent polymorphs, that is, a low-temperature orthorhombic and a high-temperature monoclinic structure [42].

Research on electrocatalytic ammonia synthesis has made significant progress in addressing challenges associated with molecular–catalyst mechanisms, leading to improvements in FE and enabling the full realization of the NRR. However, certain issues remain unresolved, including low generation rates and inadequate material characterization technologies. NRR still has a long way to go [19].

### 2.2. Chemical Bond Breaking and Directional Coupling Mechanism in Electrocatalytic N_2_ Reduction Reaction

In general, NRR can be expressed by the reaction formula:

N_2_ + 6H^+^ + 6e^−^ → 2NH_3_

The widely accepted pathways for the synthesis of NH_3_ through the reduction of N_2_ on the catalyst surface can generally be divided into two categories: the dissociation pathway and the association pathway. In the dissociation pathway, first, N≡N is directly cleaved to produce two isolated N atoms on the substrate surface; then, the two isolated N atoms are hydrogenated to produce two NH_3_ molecules. The associative pathway can be divided into distal, alternating, and enzymatic mechanisms based on the adsorption configurations of N_2_. (1) The distal pathway: N_2_ molecules are adsorbed on the substrate in a terminal configuration. Protonation and reduction occur first at the most distal N atom; after three steps of protonation and reduction, NH_3_ is successfully synthesized. (2) The alternation pathway: N_2_ is adsorbed on the substrate in a terminated configuration. First, protonation and reduction take place at the furthest N atom; then, these reactions alternate to form the *NH_2_NH_2_ intermediate; finally, the *NH_2_NH_2_ intermediate is protonated and reduced sequentially, forming and releasing two equivalents of NH_3_. (3) The enzyme pathway: N_2_ is adsorbed on the substrate in a lateral configuration. Two of the N atoms bind to the substrate surface; protonation and reduction occur sequentially to form the *NH_2_NH_2_ intermediate, which is further protonated to produce NH_3_ [32]. However, due to the extremely high bond energy of the stable N≡N bond (948 kJ mol^−1^), activating N_2_ is very challenging in electrochemical systems. To develop a suitable catalyst with the ability to cleave the N≡N bond and improve the efficiency of electrocatalytic ammonia synthesis, scientists have conducted experimental studies [43,44].

#### 2.2.1. N_2_ Conversion Using Perovskite and Non-Thermal Plasma

The studies found that perovskite is particularly suitable for oxidation reactions, including the oxidation of nitrogen oxides and carbon monoxide [45,46,47]. Additionally, non-thermal plasma (NTP: a gas comprising numerous high-energy electrons at a low temperature) promotes the activation of N_2_ under mild conditions [48]. Based on these two findings, scientists have proposed a new strategy to address the challenge of breaking the N≡N bond: using LaFeO_3_ as a catalyst, N_2_ and O_2_ in the air undergo oxidation to form NO_x_ in NTP. Subsequently, NO_x_ species react with H_2_O to generate NO_3_^−^ and NO_2_^−^. Finally, NH_3_ is electrochemically reduced from NO_x_^−^ on a Cu/CuO catalyst.

**Figure 5:**
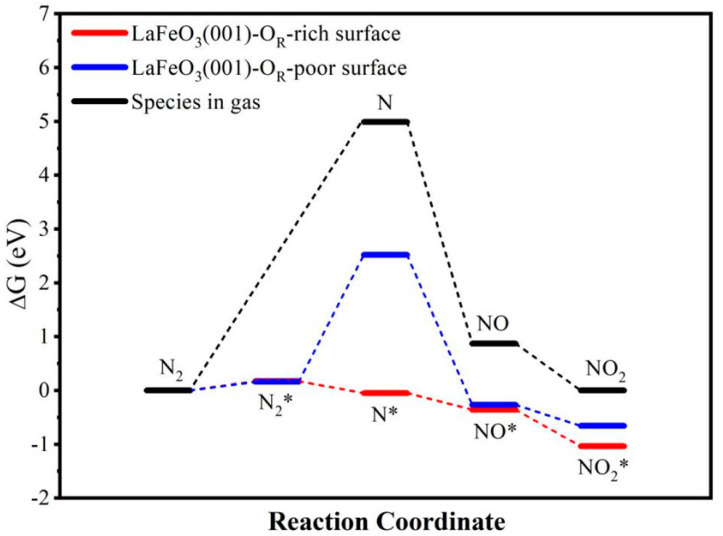
Gibbs free energy profile of N_2_ reaction on the different surfaces at 50 °C and 1 atm. * represent catalytic active sites. Copyright 2022, American Chemical Society.

The presence of the catalyst led to a significant increase in the current density in the plasma region, facilitating the activation and conversion of N_2_ and O_2_ [49,50]. To gain a deeper insight into the role of LaFeO_3_ in the atomic and molecular-level reaction process, scientists have employed theoretical calculations to assess the Gibbs free energy spectrum (ΔG) at 50 °C and 1 atm (see Figure 5). They discovered that the presence of LaFeO_3_ on both oxygen-rich and oxygen-poor catalyst surfaces substantially lowered the energy required to activate N_2_ when compared to gaseous free radicals. Thus, the presence of the catalyst surface or the catalyst itself is undoubtedly beneficial to the reaction. This strategy effectively activates N_2_ and improves the FE of the NRR, while also significantly reducing energy consumption and mitigating the energy crisis [43].

#### 2.2.2. Modification of Mo-Based Catalysts to Promote N_2_ Adsorption for Improved NH_3_ Yield and FE

**Figure 6:**
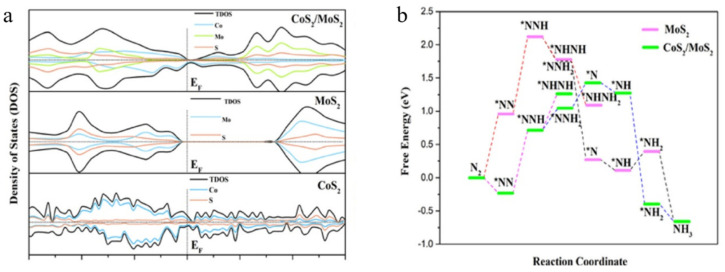
(**a**) DOS of CoS_2_/MoS_2_, MoS_2_, and CoS_2_. (**b**) Gibbs free energy diagram of the NRR on CoS_2_/MoS_2_ and MoS_2_. * represent the catalytic active sites. Copyright 2021, American Chemical Society.

Drawing inspiration from biological nitrogen fixation, scientists have developed various catalysts based on transition metal elements as the primary active centers, resembling the function of nitrogenase [51]. In this case, MoS_2_ is used as an example. The research indicates that, on the one hand, MoS_2_ is a graphene-like layered material with a tunable electronic structure and is abundant in nature [52]. On the other hand, the presence of electron-deficient/electron-rich regions is an indispensable property for the catalyst to effectively adsorb N_2_ and destroy the N≡N bond, respectively [53]. If it is possible to introduce a disulfide with a metal atom with a large difference in the electron affinity from the Mo as the central atom to form a strong interaction with the MoS_2_, it is feasible to create electrophilic and nucleophilic regions through charge modulation. FeS_2_ and MoS_2_ possess different Fermi energy levels. When they were integrated into an FeS_2_/MoS_2_ heterojunction, the formed interface can stimulate spontaneous charge transfer and then generate a special space–charge region to drive the targeted surface reaction. The band bending at the interface facilitated the charge redistribution, until the electrocatalytic system reached a thermal equilibrium state. Then, the oppositely charged regions arise at the heterointerface and bring about the alteration of the electron density around the interface, which was favorable for the targeted adsorption and activation of inert N_2_ molecules. This approach aims to enhance the effective adsorption of N_2_ and promote the breaking of N≡N bonds [51]. Additionally, some cases such as MoS_2_, SnS_2_, and CoS_2_ were also elucidated in detail to reveal conformational relationships. Due to cobalt’s lower electron affinity compared to molybdenum, the electrons are transferred from CoS_2_ to MoS_2_ during bonding. This results in an electron-deficient region near the CoS_2_ side, which can accept the lone pair electrons of the N_2_ molecules, aiding in the N_2_ absorption [54,55]. Conversely, a nucleophilic region forms near the MoS_2_ side, accumulating a significant charge that can provide electrons to the empty antibonding orbital of N_2_ and facilitate the cleavage of the N≡N bonds [56]. To test this hypothesis, scientists fabricated nanocomposites of CoS_2_/MoS_2_, which exhibited excellent catalytic activity against the NRR, as anticipated. Following the experiment, the researchers conducted density functional theory (DFT) calculations (Figure 6) to further investigate the catalytic mechanism, with the results once again confirming the hypothesis [57].

Before officially undertaking the experiment on the electrocatalytic nitrogen synthesis of ammonia reaction, the scientists initially compared the vibration modes of the CoS_2_/MoS_2_ nanocomposites with pure MoS_2_ and pure CoS_2_, respectively. They discovered that the nanocomposite caused the Mo-S bond to soften and reduced the vibration frequency of Mo-S, thus confirming the strong interaction between CoS_2_ and MoS_2_ [58,59]. Subsequently, using the CoS_2_/MoS_2_ nanocomposites as catalysts for the NRR, the scientists conducted experiments. The experimental data demonstrated that the charge transfer resistance of CoS_2_/MoS_2_ (2.9 Ω) was slightly smaller than that of CoS_2_ (5.6 Ω), but significantly smaller than that of MoS_2_ (177.4 Ω), suggesting that the interface between CoS_2_ and MoS_2_ effectively regulated the electronic structure, accelerated the electron transfer process, and promoted the reaction kinetics. Additionally, the double-layer capacitance of CoS_2_/MoS_2_ (26.8 mF cm^−2^) was substantially higher than that of CoS_2_ (1.9 mF cm^−2^) and MoS_2_ (2.8 mF cm^−2^), indicating that the introduction of CoS_2_ provided more electrochemically active sites. Furthermore, the evident decrease in the activation energy for the N_2_ reaction on the Gibbs free energy diagram (Figure 6) indicated that the CoS_2_/MoS_2_ nanocomposite exhibited superior catalytic performance for the NRR compared to pure CoS_2_ and MoS_2_. Notably, no by-products such as N_2_H_4_ were detected during the experiment, further confirming that the CoS_2_/MoS_2_ catalyst displayed high selectivity for NH_3_ formation, which was the objective of developing the new catalyst [52,57,60,61].

#### 2.2.3. Boron, Carbon, and Nitrogen Cooperate with the Nanotube Single Atom during Electrocatalytic Reduction of Nitrogen to Ammonia

At present, single-atom catalysts (SACs) have demonstrated significant advantages in various catalytic reactions. Compared to traditional catalysts, SACs can greatly increase specific activity and reduce noble metal loading. Previous studies have shown that by manipulating the electronic structure of SACs, the catalytic activity and selectivity can be modified [62,63,64,65].

**Figure 7:**
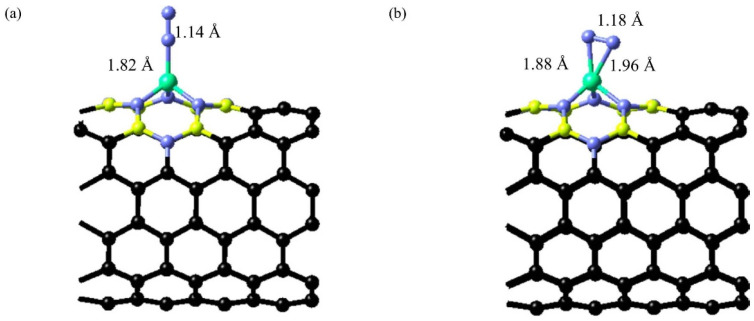
The relaxed configuration of N_2_ in the (**a**) end-orientated mode; (**b**) side-orientated mode on a Mn-embedded BCN NT. Black, yellow, violet and green color represent the C, B, N and Mn stoms. Copyright 2022, Elsevier.

There are two known configurations of N_2_ surface adsorption: end-oriented and side-oriented. Experimental results indicate that the side-oriented mode is more resilient to N–N bond elongation. On the other hand, the end-oriented mode exhibits greater adsorbed energy. Consequently, the end-oriented mode is more favorable for stable adsorption and complete activation of N_2_. In this study, structurally optimized Mn is embedded in boron–carbon–nitrogen nanotubes (BCN NTs), enabling the Mn atom to bind with the three surrounding N atoms and securely adsorb to the substrate surface [63,66]. In the end-oriented mode, the Mn–N bond measures 1.82 Å, whereas in the side-oriented mode, two Mn–N bonds occur with lengths of 1.88 Å and 1.96 Å, respectively. Post-binding, the N–N bond extends to 1.14 Å (in the terminal mode) and 1.18 Å (in the lateral mode) from its initial 1.12 Å length, indicating a tendency for N≡N to break and favor the formation of NH_3_ (see Figure 7) [67]. In simpler terms, when N_2_ is adsorbed onto the Mn atom in the terminal mode, the N≡N bond length increases, making it easier to break. Additionally, the hybridization of the d orbital of the transition metal (TM) with the antibond π* of N_2_ can create a low-energy system, significantly reducing the energy barrier for the reaction. Consequently, MN-embedded BCN NTs have been further validated as a promising catalytic material.

## 3. Electrocatalytic NO_3_^−^ Synthesis of Ammonia (NO_3_^−^RR)

### 3.1. Mechanism of Molecular–Catalyst Interaction for Electrocatalytic NO_3_^−^ Reduction Reaction

During the process of NRR, it has been observed by scientists that the fracture of N≡N requires an extremely high amount of energy, and the solubility of N_2_ in water is quite low. As a result, the NRR typically exhibits low reaction rates and FE. In order to address this issue, scientists are actively searching for alternative nitrogen sources that possess better properties compared to N_2_ for electrocatalytic ammonia synthesis. One nitrogen source that has garnered significant attention from researchers is NO_3_^−^. This is primarily due to its high solubility in water and the low bond energy of N–O (requiring only 21.7% of the energy needed for the dissociation of N≡N) [43]. Furthermore, the electrocatalysis of NO_3_^−^ ammonia synthesis offers a potential solution to the problem of water pollution caused by the use of nitrogenous fertilizers and the discharge of industrial wastewater. Additionally, the analysis of the thermodynamics and kinetics of this reaction has revealed its potential to reduce energy consumption and alleviate the energy crisis to some extent [68].

The NO_3_^−^RR starts with the adsorption of NO_3_^−^ on the catalyst surface, which is then reduced to the intermediate product, NO_2_. This NO_2_ absorbs more charge and, subsequently, decomposes into NO and N. Through a series of continuous deoxidation and hydrogenation reactions, OH^−^ is produced in water, and NH_4_^+^ and NH_3_ are formed. The overall reaction can be summarized as follows: NO_3_^−^ + 6H_2_O + 8e^−^ → NH_3_ + 9OH^−^. It is worth noting that the intermediate NO_2_ plays a crucial role in the electrochemical reduction of NO_3_^−^ to ammonia. However, its N–O bond energy is relatively large and difficult to break, making it a limiting step in the overall reaction rate when catalyzed by certain catalysts. Subsequently, NO_2_ absorbs more charge and breaks down into NO and N. Nevertheless, the intermediate products NO and N_2_O may detach and form by-products, such as NO and N_2_O, along with several other possible reaction pathways (NO_2_, NO_2_^−^, N_2_, NH_2_OH, NH_3_, and N_2_H_4_), thereby significantly reducing the selectivity of NO_3_^−^RR. It is evident that the hydrogen evolution reaction resulting from the H* combination is the most prevalent competing reaction for NO_3_^−^RR [69,70,71]. By carefully selecting an appropriate catalyst, altering the structural characterization of the catalyst, or developing a new catalyst that allows for specific NO_3_^−^ adsorption, it becomes possible to enhance the selectivity of NO_3_^−^RR, reduce the production of other undesired products, and improve the reaction activity. Scientists have made considerable progress in developing various types of catalysts, and this section will provide a summary of the four most significant ones: precious metal catalysts, non-precious metal catalysts, metal oxide catalysts, and metal-free catalysts [72].

#### 3.1.1. Noble Metal Catalyst

Noble metals like Au, Pd, Pt, and Ru exhibit excellent catalytic activity for the NO_3_^−^RR reaction. However, considering the high cost of noble metals, it is important to optimize the atomic utilization. As a result, the most widely studied catalysts for noble metals are monatomic species, nanostructures, and alloys [73,74]. Among noble metals, ruthenium (Ru) is commonly used for the electrocatalytic reduction of nitrate to ammonia. For instance, Li et al. developed a strained Ru nanocluster catalyst that efficiently reduces nitrate to ammonia at room temperature, exhibiting rapid kinetics, high selectivity, and strong current density. The catalyst’s high-level performance is attributed to the presence of the tensile lattice strain, which enhances the H–H coupling barrier and suppresses the hydrogen evolution reaction (HER), while also facilitating the production of NH_3_ through efficient H* generation. Consequently, the strain nanostructures demonstrate exceptional ammonia production rates and maintain high selectivity over a broad range of operational potentials [25,75,76,77,78]. However, due to the characteristics of noble metals, such as their scarcity and high cost, their practical implementation is greatly limited. In contrast, non-noble metals are abundant and cost effective, which makes them easier to put into practice in production processes.

#### 3.1.2. Non-Noble Metal Catalysts

Non-noble metal catalysts have high selectivity for the NO_3_^−^RR reaction and can synthesize ammonia cheaply, efficiently, and sustainably. Researchers have extensively investigated highly efficient non-noble metal catalysts for the electrochemical NO_3_^−^RR reaction [79]. The following discussion focuses on examples using Cu and Fe catalysts [80].

##### Cu-Based Electrocatalyst Reacts in the Nitrate Reduction Reaction (NO_x_^−^RR)

**Figure 8:**
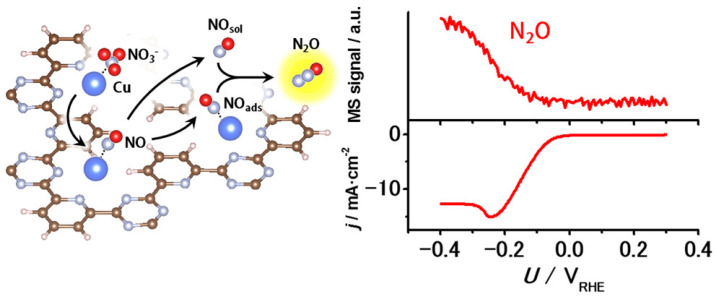
The path diagram of the copper-modified covalent triazine skeleton (Cu-CTF) catalyzed nitrate electrochemical reduction and the results of the Cu-CTF density functional calculation. Copyright 2016, American Chemical Society.

Metallic Cu-based materials are the most widely studied electrocatalysts for the NO_3_^−^RR reaction due to their favorable NO_3_^−^ adsorption, high FE at a low current density, excellent selectivity, effective HER inhibition, and low cost. The Cu catalyst undergoes restructuring during NO_3_^−^RR electrocatalysis, with the extent depending on the Cu loading and reaction potential. Under negative reaction potentials, Cu monoatomic sites readily aggregate, transforming into Cu clusters and nanoparticles. These reconstructed Cu species can reversibly transform back to Cu monoatoms through peroxidation-driven redispersion under environmental conditions. The reconstructed form of copper significantly enhances the rate of ammonia reduction. For instance, experiments have demonstrated the effectiveness of the copper-modified covalent triazine skeleton (Cu-CTF) as an electrocatalyst for the reduction of nitrate, as depicted in Figure 8 [81]. Nevertheless, the cumulative impact of nitrite (NO_2_^−^) on copper, along with the recombination of highly active Cu-based electrocatalyst during the NO_3_^−^RR reaction, makes it challenging to identify the dynamic active sites and conduct comprehensive studies on the catalytic mechanism. Consequently, selective NH_3_ production using copper as an electrocatalyst remains unadvisable [82,83,84,85,86]. Compared with other non-noble metal catalysts, Cu-based electrocatalysts are more easily used in actual production.

##### Fe-Based Electrocatalysts for NO_3_^−^RR

**Figure 9:**
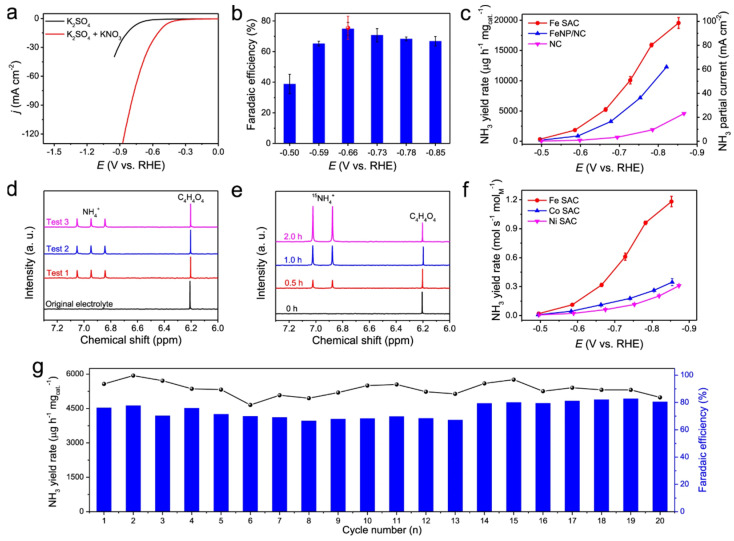
(**a**) Linear sweep voltammetry curves for the Fe SAC in K_2_SO_4_ electrolyte and KNO_3_/K_2_SO_4_ mixed electrolyte. (**b**) NH_3_ FE for Fe SAC at each given potential. Red dot is FE estimated by three independent NMR tests. (**c**) NH_3_ yield rate and partial current density for Fe SAC, FeNP/NC, and NC. (**d**) Nuclear magnetic resonance hydrogen spectra for the electrolytes after three independent nitrate reduction tests. (**e**) Nuclear magnetic resonance hydrogen spectra of electrolyte after ^15^NO_3_^−^ reduction test with K^15^NO_3_/K_2_SO_4_ mixed electrolyte at different times. (**f**) NH_3_ yield rate for Fe SAC, Co SAC, and Ni SAC based on metal content. (**g**) The cycling tests for Fe SAC. Catalyst loading for all of the electrocatalytic nitrate reduction tests is the same. Copyright 2021, Nature.

Fe-based monatomic catalysts are synthesized using a TM-assisted carbonization method and SiO_2_ powder as a hard template. The catalytic performance of these catalysts in electrochemical NO_3_^−^RR is primarily dependent on the activity of iron active sites and the number of available active sites. Nitrogen is uniformly dispersed in order to coordinate the positive charge introduced by the metal sites, and it has a moderate interaction with NO_3_^−^, resulting in excellent NO_3_^−^RR performance. Analysis of the X-ray spectra reveals that the oxidation state of Fe single atoms lies between Fe^2+^ and Fe^3+^. Delocalized electrons of Fe single atoms can be shared by porphyrin-like structures, which enhances the catalyst’s conductivity while reducing its nitrate catalytic activity. Experiments conducted by Wu indicate that the concentration of NO_3_^−^ has no significant effect on the selectivity of NH_3_ on Fe-based monatomic catalysts. However, the content of NO_2_^−^ generation decreases with increasing iron-based monatomic catalysts and the electric current, thereby leading to an increase in NH_3_ selectivity (as shown in Figure 9) [87,88,89,90,91,92]. However, due to the high uncertainty of the active site, further research is still needed.

#### 3.1.3. Metal Oxide Catalyst

**Figure 10:**
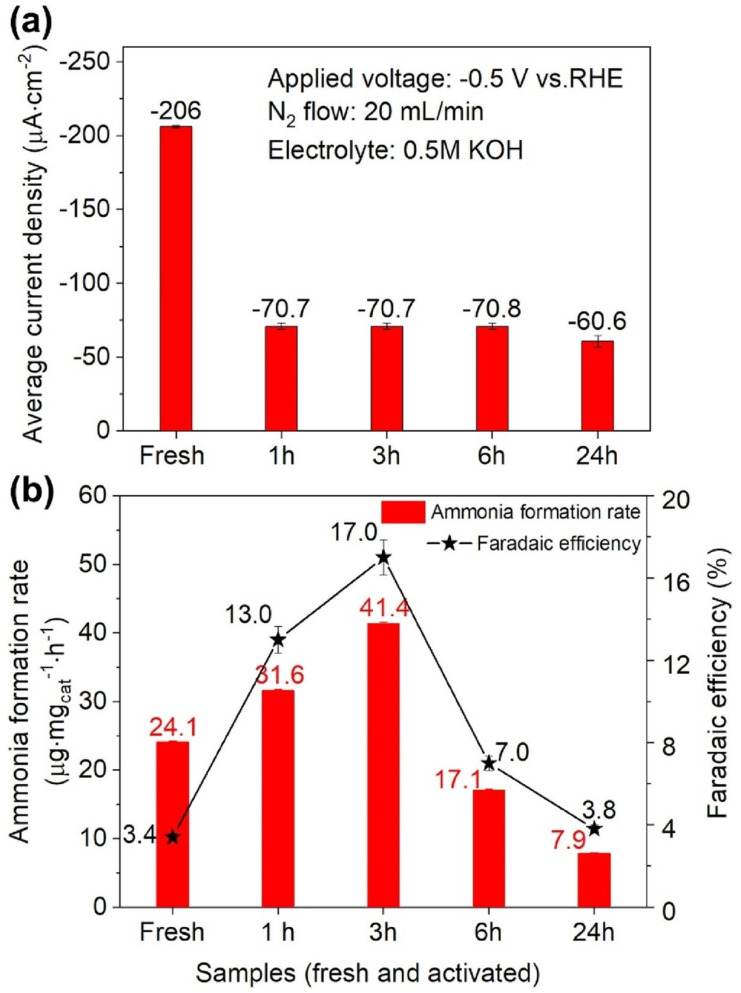
(**a**) Sample current density before and after activation. (**b**) Ammonia formation rate and Faradaic efficiency. Copyright 2020, Elsevier.

Transition metal oxides have several advantages, such as natural abundance, ecological friendliness, and chemical stability, and are suitable as catalysts to catalyze reactions. Among them, Cu, Co, Ag, Ti, and Ru, primarily form oxides. These oxides exhibit various structures and phases, including defects and oxygen vacancies. These oxides effectively catalyze nitrate ions to ammonia, exhibiting outstanding nitrate conversion efficiency, a significant ammonia yield or conversion, high FE, and strong ammonia selectivity [93,94]. One example is the CuO catalyst, composed of copper oxide (CuO). CuO nanowire arrays exhibit an impressive FE (95.8%) and high ammonia selectivity (81.2%). They serve as highly efficient cathode materials for electrocatalyzing the reduction of nitrate ions to ammonia [84,95,96]. Another example is iron oxide, including Fe_2_O_3_ and Fe_3_O_4_. Activation of the sample with Fe_2_O_3_ single-bond carbon nanotubes resulted in a substantial decrease in the current density. However, the NH_3_ generation rate remained high, suggesting a change in the catalyst’s performance during activation. This change not only suppressed HER competition, but also enhanced the FE of NH_3_ (Figure 10) [97]. In summary, transition metal oxide catalysts have excellent stability, but there are still some difficulties in practical implementation.

#### 3.1.4. Metal-Free Catalyst

Compared to metal catalysts, metal-free catalysts offer several advantages, including lower costs, greater environmental friendliness, and reduced toxicity. In summary, the development and exploration of metal-free catalysts holds significant scientific importance and economic value. Currently, carbon-based catalysts are predominantly reported as electrochemical NRR metal-free catalysts. These catalysts function by disrupting the homogeneous charge density of carbon atoms through the introduction of foreign atoms with different electronegativity and atomic radius. This process leads to the formation of more free electrons in the delocalized π orbital of the carbon skeleton, thereby enhancing the catalytic activity [91,98,99,100,101].

**Figure 11:**
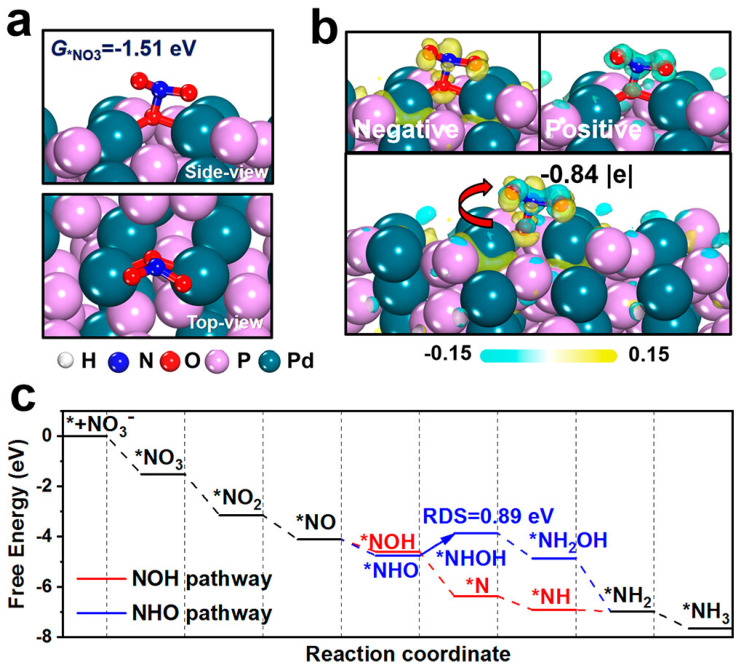
(**a**) Configurations of *NO_3_ on PdP_2_. (**b**) Differential charge-density map of *NO_3_ on PdP_2_. Yellow and cyan denote charge accumulation and depletion, respectively. * represent the catalytic active sites. (**c**) Adsorption free energy diagrams of PdP_2_ and the corresponding intermediates. Copyright 2023, American Chemical Society.

To exemplify the impact of a metal-free catalyst on the electrocatalytic reduction of ammonia with nitrate, we will analyze the reduction of graphene oxide. Wang and his team investigated the electrocatalytic reduction of nitrate to ammonia mechanism (NRA) of PdP_2_ nanoparticles on reduced graphene oxide (PdP_2_/RGO) [102]. Initially, they examined the adsorption of three NO_3_^−^ geometries on the PdP_2_ surface: top Pd site 1, top Pd site 2, and bridge Pd site. Comparative analysis of the adsorption free energy of NO_3_^−^ at different sites revealed a preference for NO_3_^−^ absorption at the bridge Pd site (Figure 11) [102]. Furthermore, the analysis of the partial density of the state indicated significant hybridization between NO_3_^−^ p and Pd d orbitals, demonstrating a strong interaction between Pd and *NO_3_^−^. This analysis suggests that the PdP_2_ surface effectively activates and hydrogenates NO_3_^−^, while inhibiting the competing HER [103].

### 3.2. Chemical Bond Breaking and Directional Coupling Mechanism in Electrocatalytic NO_3_^−^ Reduction Reaction

**Figure 12:**
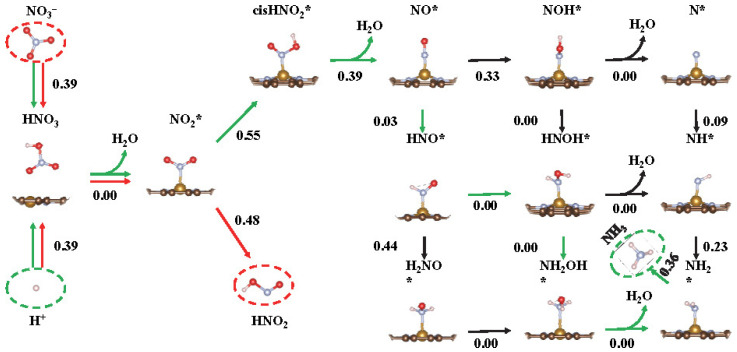
Kinetic barriers on FeN_4_ to NH_3_ and HNO_2_ at −0.50 V vs. RHE. The pathways to produce NH_3_ and HNO_2_ are highlighted with green and red lines, respectively. * represent the catalytic active sites. The pink, red, light blue, brown, and bright yellow represent H, O, N, C, and Fe atoms, respectively. Copyright 2023, Elsevier.

The electrocatalytic NO_3_^−^ reduction reaction involves eight electron transfers and nine proton transfers, presenting a significant challenge for reaction selectivity [68]. As an illustration, the selectivity between nitrite and ammonia gas is examined to explain the directional coupling mechanism in the electrocatalytic ammonia synthesis from nitrate. Incomplete conversion of nitrates can result in the formation of nitrite (NO_2_^−^), which can cause liver damage, hyperhemoglobinemia, and potential cancer development in humans. The formation of HNO_2_ is facilitated by overlapping the reaction steps with NH_3_ production. Analysis of the kinetic barriers reveals that the protonation of NO_2_ to HNO_2_ is crucial for determining product selectivity. At −0.50 V vs. RHE, the protonation of NO_2_* to HNO_2_ exhibits a lower potential barrier (0.48 eV) compared to the highest barrier for NH_3_ formation (0.55 eV) (Figure 12). Subsequent investigations involving microdynamics modeling, electronic structure analysis, and consistent energy barrier changes, provided further evidence that the difference in the protonation barriers between NO_2_ and HNO_2_ or cisHNO_2_ is crucial for determining product selectivity. Moreover, due to the smaller charge transfer coefficient for the protonation of NO_2_* to HNO_2_ at higher overpotentials, the production of HNO_2_ is favored. Existing experimental data indicates that FeN_4_ exhibits superior catalytic performance compared to other catalysts, including CuN_4_, NiN_4_, and CoN_4_, and promotes the NH_3_ generation pathway. Moreover, NO_3_^−^RR produces NH_3_ via the pathway on FeN_4_ (R4: NO_2_→cisHNO_2_) and HNO_2_ generation (R7: NO_2_→HNO_2_) is nearly equal, whereas the free energy of HNO_2_ formation (R7: NO_2_→HNO_2_) for the other three catalysts (CuN_4_, NiN_4_, CoN_4_) is lower than that of the NH_3_ selection step (R10: NO→HNO), highlighting a preference for HNO_2_ production. The discovery of FeN_4_ offers valuable insights for the development of novel and enhanced catalysts, prompting researchers to conduct further experimentation and design catalysts that align with this concept [104].

## 4. Conclusions and Perspectives

In order to help readers understand the advantages of the various catalysts listed in this review, some examples of the different types of catalysts for the two reactants mentioned in the text have been compiled in this review, which aim to show readers in a clear and concise manner the effects of different catalysts on ammonia yield and FE in the electrocatalytic ammonia synthesis reaction (Table 1 and Table 2).

Researchers have extensively explored various methods and developed numerous catalysts to address issues related to low selectivity for NH_3_ and high energy consumption during the NRR and NO_3_^−^RR processes. In existing studies, transition metals have always played an important role in ammonia synthesis. Nonetheless, challenges persist, including the high cost of catalysts and the inability to completely eliminate competitive reactions. In 2018, scientists used the concept of main-group metal mimetization to speculate that the NRR can be made efficient under transition metal-free conditions. Materials such as carbene, di-coordinated boron olefins, and others were later shown to be able to cleave the N≡N bond by repeating reduction–protonation steps from an end-on bridging N_2_ complex, successfully proving the scientists’ conjecture, and in the future, non-transition-metal catalysts may also become an emerging research hotspot [105].

## Figures and Tables

**Figure 2:**
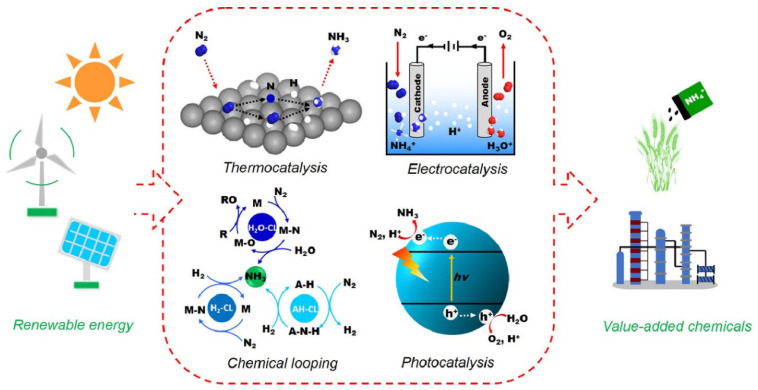
Diagram of thermal catalysis, electrocatalysis, photocatalysis, and chemical cyclization processes for the synthesis of NH_3_ from renewable energy sources. Copyright 2019, Elsevier.

**Table 1:** Summary of electrocatalysts for the reduction of N_2_ to NH_3_.

Catalyst Category	Catalyst	Reactant	Electrolyte	Potential (V vs. RHE)	Ammonia Yield	FE	Ref.
metal complex electrocatalyst	MoS_2_	N_2_	0.1 M Na_2_SO_4_	−0.5	5.39 μg cm^−2^ h^−1^	1.17%	[29]
	ZnS	N_2_	__	−0.1	7.1 × 10^6^ mol s^−1^ cm^−2^	0.964%	[29]
	CoS_2_/MoS_2_ nanocomposite	N_2_	__	__	54.7 μg mg_cat_^−1^ h^−1^	20.8%	[54]
Metal-free catalyst	N-doped carbon	N_2_	__	−0.9	3.88889 × 10^−16^ mol s^−1^ mg^−1^	1.42%	[29]
	PCN-NVs	N_2_	0.1 M HCl	−0.2	8.09 μg mg_cat_^−1^ h^−1^	11.59%	[29]
Non-noble metal electrocatalyst	Mo nanofilm	N_2_	__	−0.29	3.09 × 10^−11^ mol s^−1^ mg^−1^	0.72%	[29]
	Porous Ni	N_2_	0.01 M H_2_SO_4_	−3.6	0.998 μg cm^−2^ h^−1^	0.89%	[29]
Noble metal electrocatalyst	Ru SAs/g-C_3_N_4_	N_2_	0.1 M NaOH	0.05	23.0 μg mg_cat_^−1^ h^−1^	8.3%	[29]
	Ru-C	N_2_	2 M KOH	−1.1	0.21 μg cm^−2^ h^−1^	0.28%	[29]
	Rh ultrathin nanosheets	N_2_	0.1 M KOH	−0.2	23.88 μg mg_cat_^−1^ h^−1^	0.217%	[29]
Transition metal catalyst	NiTe nanocrystals with {001} surface exposure	N_2_	__	−0.1	33.34 ± 0.70 μg mg^−1^ h^−1^	17.38 ± 0.36%	[31]
	NiTe nanocrystals with {010} surface exposure	N_2_	__	−0.1	12.78 ± 0.43 μg mg^−1^ h^−1^	8.56 ± 0.22%	[31]
Single-Atom Catalyst	Ru	N_2_	—	−0.42 (V vs. SHE)	__	97%	[60]
	Rh	N_2_		−0.47 (V vs. SHE)	__	73%	[60]

**Table 2:** Summary of electrocatalysts for the reduction of NO_3_^−^ to NH_3_.

Catalyst Category	Catalyst	Reactant	Electrolyte	Potential (V vs. RHE)	Ammonia Yield	FE	Ref.
Noble metal catalyst	Pd-NDs/Zr-MOF	NO_3_^−^	__	−1.3	287.5 mmolNH_3_ h^−1^ g_cat_^−1^	60%	[70]
Noble metal catalyst	Ag-NDs/Zr-MOF	NO_3_^−^	__	−13	275 mmolNH_3_ h^−1^ g_cat_^−1^	55%	[70]
Monometallic Catalyst	Cu	NO_3_^−^	__	−0.15	390.1 μg mg_Cu_^–1^ h^–1^	99.7%	[77]
Monometallic Catalyst	Fe	NO_3_^−^	__	−0.85	~20,000 μg h^−1^ mg_cat_^−1^	~66%	[84]
metal oxide catalyst	Fe_2_O_3_-CNT	NO_3_^−^	0.5 M KOH	−0.5	41.4 μg h^−1^ mg_cat_^−1^	17%	[94]

## Data Availability

Date are contained within the article.
